# Bacterial Communities as Modulators of Innate Immune Signalling: An In Vitro Perspective on Toll‐Like Receptor Activation

**DOI:** 10.1111/1758-2229.70289

**Published:** 2026-02-04

**Authors:** Elke Eriksen, Pål Graff, Anani Komlavi Afanou

**Affiliations:** ^1^ STAMI, National Institute of Occupational Health, Research Group for Chemical Work Environment Oslo Norway; ^2^ STAMI, National Institute of Occupational Health, Research Group for Occupational Toxicology Oslo Norway

**Keywords:** bacteria, high throughput sequencing, toll‐like receptors, work environment

## Abstract

Investigating the work‐environmental microbiome is critical for assessing occupational risk associated with exposure to microorganisms. The present study examined the bacterial composition of inhalable dust from waste sorting plants and explored their potential to induce Toll‐like receptors (TLR) in vitro, thereby providing insights into the immunomodulatory potential of complex microbial communities from occupational settings. These findings highlight how few dominant bacterial species shape the immune activation properties of the overall bacterial community, where less abundant taxa play a crucial role in immune modulation through TLR activation. The strong association between TLR activation and rare yet highly inductive bacterial taxa demonstrates their potential immunological significance, suggesting that even low‐abundant microbes may have a disproportionate impact on immune responses and occupational health outcomes.

## Graphical abstract

Bacterial contaminants in occupational environments may elicit an immune response. This study investigated the immune‐modulatory potential of bacterial communities using in vitro cell‐based assays and revealed distinctive bacterial species as drivers of TLR2 and TLR4 activation. The presented approach studied the role of bacterial species as modulators of TLR‐mediated innate immune response and thereby contributed to a better understanding of the potential health implications of workers in modern waste management.

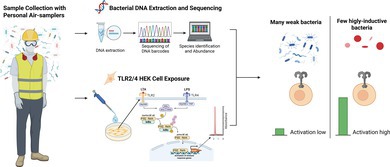

## 1 Introduction

Exposure to microorganisms is a significant concern in occupational settings such as agriculture (Molina‐Guzmán and Ríos‐Osorio 2020), waste management (Madsen et al. 2021) and healthcare (Monteiro et al. 2022) due to their potential to disturb immunological homeostasis. Upon inhalation or ingestion, these microorganisms may interact with the host's immune system, eliciting both innate and adaptive immune responses. Previous research has identified waste sorting plants as significant sources of occupational exposure to microorganisms, especially in the presence of organic material (Szulc et al. 2022; Eriksen, Afanou, Straumfors, et al. 2023). However, as microbial communities vary greatly between and within workplaces (Eriksen, Afanou, Madsen, et al. 2023), the pathogenic potential and the likelihood of triggering immune responses may be divergent in different occupational exposure scenarios. In fact, Afanou and colleagues identified negative associations between fungal richness and TLR activation, despite positive correlations with fungal spore levels contained in the total dust fraction (Afanou et al. 2023). They concluded on divergent relationships between fungal spore levels and species richness towards immunological responses and emphasised the need for a thorough investigation of the microbiome in order to identify hazardous components with immune‐stimulatory potential that may be encountered in the work‐environment.

The associations between immunological responses and work exposure are complex and challenging to study in vivo. As alternative approaches, cell models expressing specific Toll‐like receptors (TLR) upon exposure to microbial ligands in vitro are used *(*Radakovics et al. 2021 *)*. TLRs are expressed on various cell types, including monocytes, macrophages, dendritic cells as well as epithelial cells (Sasai and Yamamoto 2013) and are key components of the innate immune system. TLRs are involved in pathogen recognition as well as the recruitment of immune cells to the site of infection and the promotion of an inflammatory response through the secretion of pro‐inflammatory cytokines, chemokines and other mediator molecules. Furthermore, TLR activation enhances the ability of dendritic cells in their role as antigen‐presenting cells and thereby forms a link between an innate and adaptive immune response. Cell models exploit the benefits of segregating individual immunological pathways from their usual complex interplay. Recent studies have discussed the role of microbial lipoproteins to modulate TLR responses, highlighting the complexity of these interactions (Oliveira‐Nascimento et al. 2012; Xia et al. 2021). As the microbial community profile in waste sorting plants is diverse and can vary significantly based on environmental conditions and waste composition (Nguyen et al. 2021), the type and magnitude of TLR‐mediated immune activation in vitro may be highly variable between workplaces. Furthermore, previous research has shown that the immune‐stimulatory potential of microorganisms is highly erratic, and pathogenicity is often associated with single species within a genus or even single strains (Rizzetto et al. 2013; Ehrlich et al. 2008).

Despite the critical need for research on the relationship between the occupational mycobiome and innate immune responses, so far only one exposure study (Afanou et al. 2023) has investigated the fungal microbiome in waste sorting plants and its activation potential of in vitro reporter cell systems. The present study explored a similar approach to examine the associations between the bacterial microbiome and NFkB induced responses through TLR2 and TLR4 signalling in vitro, revealing that only a minor fraction of the bacterial microbiome engaged TLR receptors. These results emphasise the critical need for a holistic characterisation of complex microbial communities that may elucidate an immune response as well as provide new insights into identifying drivers of dysfunctional immunity in vitro.

## 2 Results

### 2.1 Correlation Between Diversity Indices and TLR Activation

Spearman correlation between alpha diversity estimates and TLR activation is shown in Figure S1. The Abundance‐based Coverage Estimator (ACE) was significantly and negatively correlated with TLR4 (*p* = 0.055) activation, indicating that high species richness was associated with decreased TLR activation. Divergent trends were identified for Shannon diversity. TLR2 activation was associated with low diversity (low Shannon), whereas TLR4 activation was associated with high diversity (high Shannon). None of these trends were, however, statistically significant (*p* > 0.05).

### 2.2 Taxa Inducing TLR2 and TLR4 Activation

Of a total of 1110 taxa that were identified at species level, a total of 87 (8%) of the unique taxa were significantly associated with TLR activation (*p*‐values below 0.05 were considered statistically significant and values below that were included in the analysis). Of these, 55 were significantly correlated to TLR2 activation, whereas 76 taxa were significantly correlated to TLR4 activation (Figure S2, Table S1). Forty four shared taxa were correlated with significant activation of both receptors.

**FIGURE 1:**
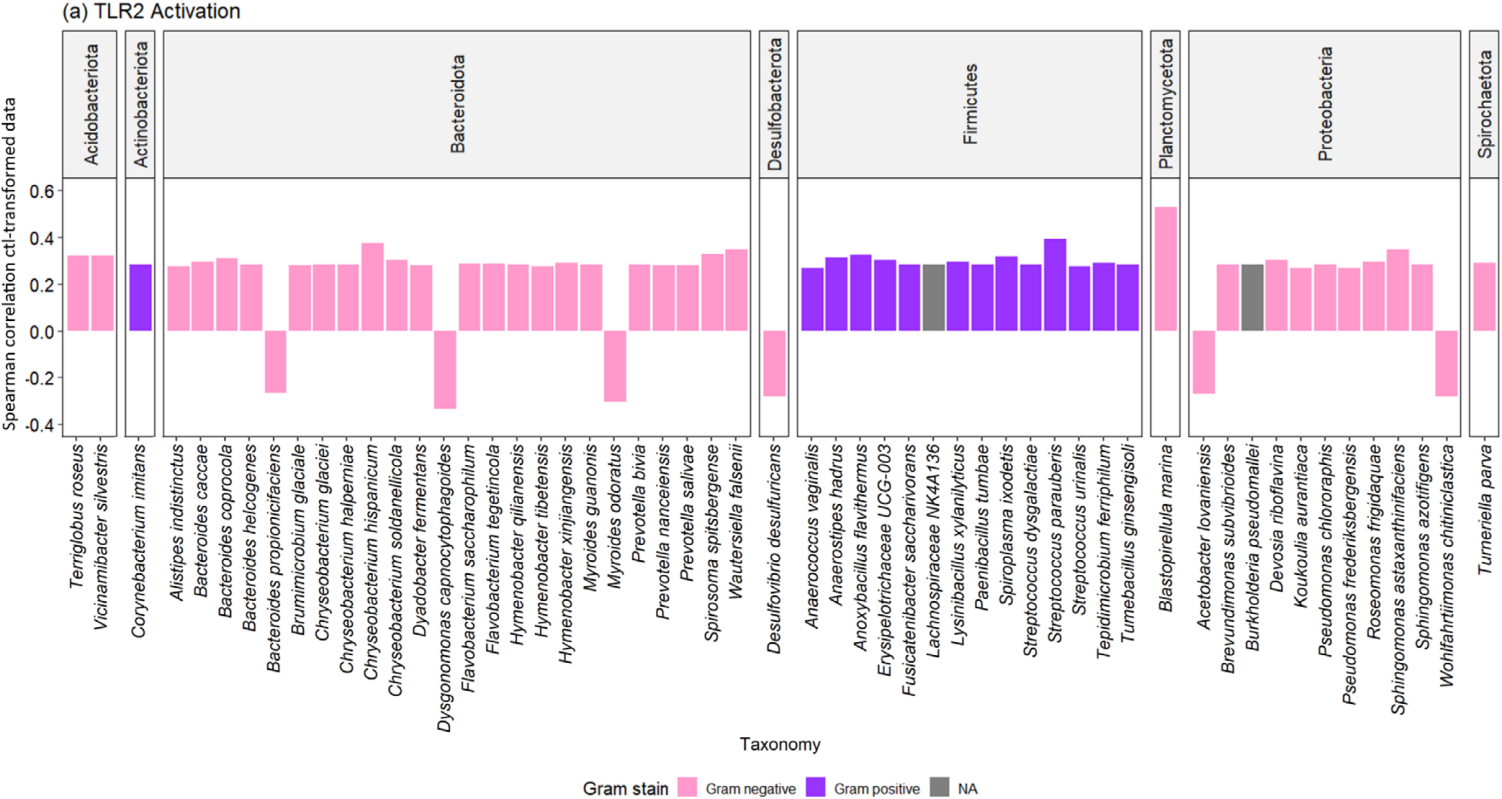
Correlation between unique taxa and TLR2 activation in vitro (Spearman correlation coefficient, *p* < 0.05). TLR2 activation in association with Gram negative (pink: 71%) and Gram positive (purple: 25%) taxa. Unknown Gram stain (grey: 4%).

Fifty five different taxa belonging to eight phyla were significantly associated with TLR2 activation in vitro (Figure 1). Of these, 24 belonged to *Bacteroidota*, 12 to *Firmicutes*, 11 to *Proteobacteria*, 2 to *Acidobacteriota* and each 1 to *Actinobacteriota*, *Desulfobacterota*, *Planctomycetota* and *Spirochaetota*, respectively. Forty nine taxa correlated positively with TLR2 activation, whereas six correlated negatively. Amongst these 55 taxa, 71% were Gram‐negative, 26% Gram‐positive and the remaining were not assigned to either. The samples contained 12 human pathogens and 25 potentially pathogenic taxa (Figure S3).

**FIGURE 2:**
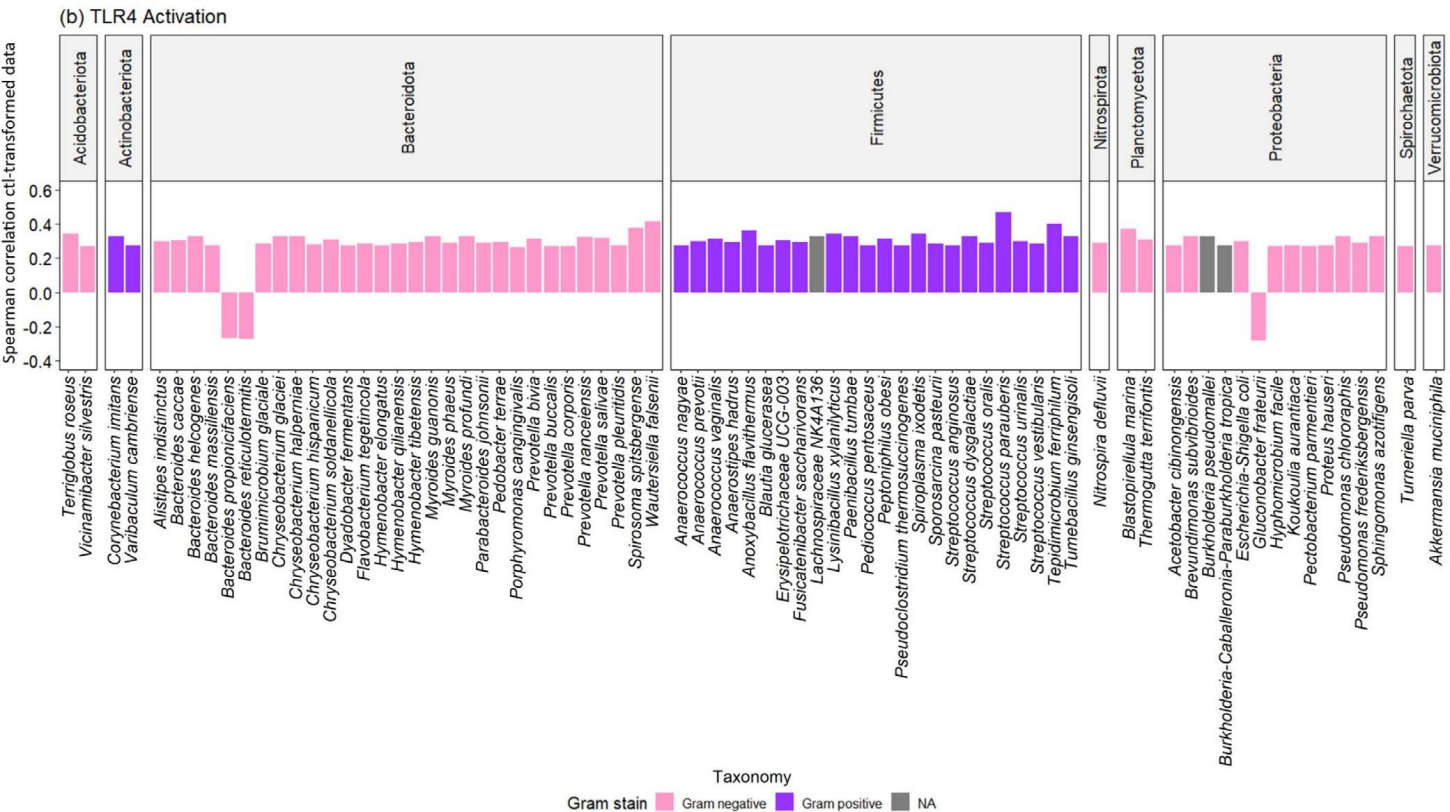
Correlation between unique taxa and TLR2 activation in vitro (Spearman correlation coefficient, *p* < 0.05). TLR4 activation in association with Gram negative (pink: 63%) and Gram positive (purple: 33%) taxa. Unknown Gram stain (grey: 4%).

A total of 76 unique taxa belonging to nine different Phyla were significantly associated with TLR4 activation (Figure 2). Of these 30 belonged to *Bacteroidota*, 24 to *Firmicutes*, 13 to *Proteobacterioa*, 2 to *Acidiobacteriota*, *Actinobacteriota* and *Planctomycetota*, respectively, as well as each 1 to *Nitrospirota*, *Spirochaetota* and *Verrucomicrobiota*, respectively. Seventy three taxa correlated positively with TLR2 activation, whereas three correlated negatively. Amongst these, 76 taxa, 63% were Gram‐negative, 33% Gram‐positive and the remaining were not assigned to either. Amongst these, 19 were human pathogens and 28 potential human pathogens (Figure S4).

### 2.3 Abundance of Significantly Correlated ASVs Stratified by TLR Receptor and Phylum

The 87 identified unique taxa consisted of 296 unique amplicon sequence variants (ASVs) that were identified at the species level. These ASVs were used to investigate the association between relative ASV abundance and TLR activation, for TLR2 (Figure S5a) and for TLR4 (Figure S5b), respectively. The slopes of the linear regressions relating relative abundance to TLR activation varied across phyla but were predominantly positive, indicating that TLR activation was associated with frequently abundant ASVs exhibiting strong inductive activity. In contrast, negative associations between relative abundance and TLR4 activation were observed in the phylum *Verrucomicrobiota*, suggesting that TLR4 activation was, on average, associated with low abundant, inductive ASVs (Table S2).

### 2.4 Abundance of Proteobacteria as Related to TLR Activation

Eleven different unique taxa were identified amongst Proteobacteria that were associated with TLR2 (Figure S6a) activation, and 13 with TLR4 activation (Figure S6b), respectively. In both cases, high TLR activation was associated with low abundant taxa that induced TLR signalling, such as *Roseomonas frigidaque* (TLR2) and *Koukoulia aurantiaca* (TLR4). In individual cases, such as *Acetobacter aceti* , divergent trends were observed, and highly abundant strains were associated with low TLR activation, and vice versa.

The 10 most abundant taxa included 133 unique ASVs and accounted for 36% of the total ASVs that were identified at species level (Figure S7).

Two different strains within the taxon *A. vaginalis* were identified (Figure S8a,b). Both were associated with significant activation of TLR2 and TLR4 receptors, respectively, despite relatively low abundance (< 0.04%) in the samples.

**FIGURE 3:**
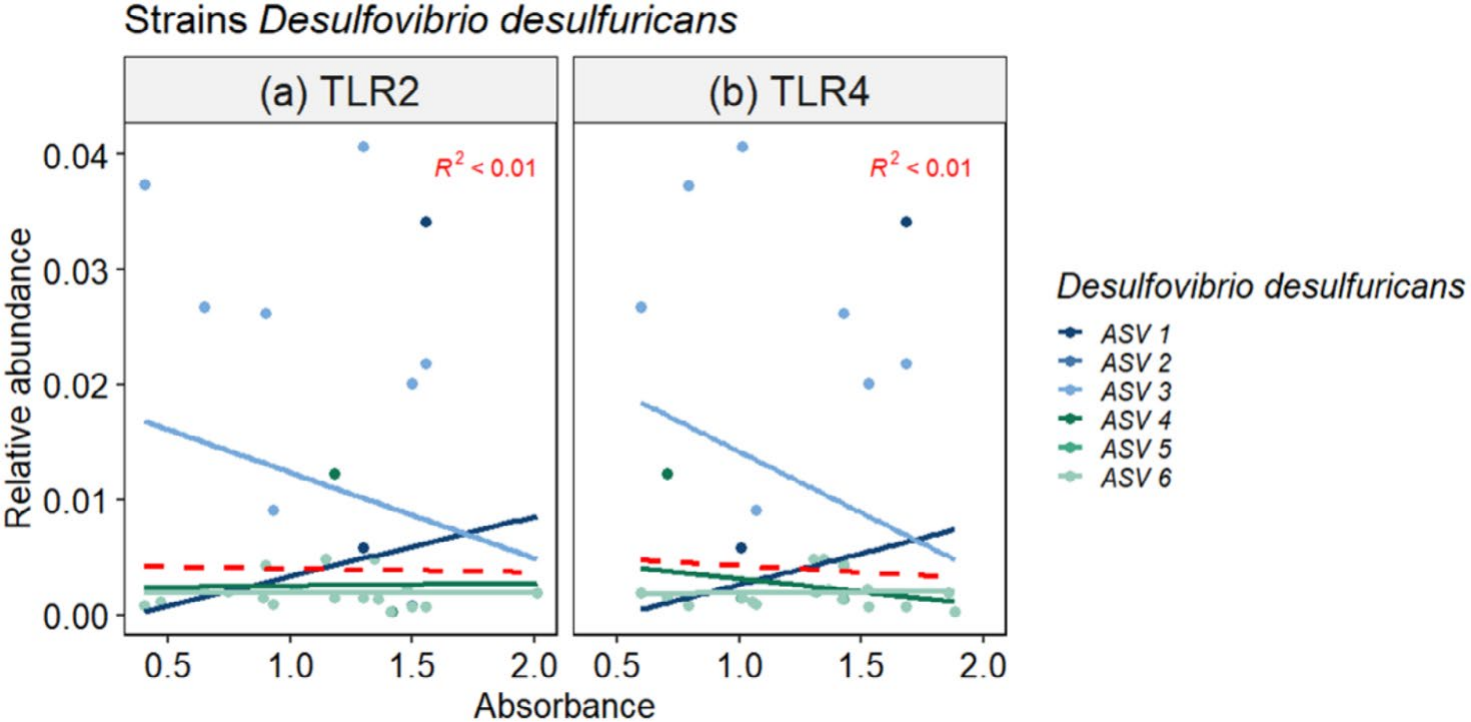
Associations between TLR activation and ASVs in the taxa *D. desulfuricans* . The graph shows samples that significantly activated TLR compared to a paternal HEK null cell line (*p* < 0.05). (a) TLR2, (b) TLR4. Regression lines indicated for individual ASVs, as well as across ASVs (red striped line) including equation of global regression line. The correlations for individual taxa are listed in Table S2.

Wihting the taxum *Desulfovibrio desulfuricans* six different strains with varying association to TLR activation were identified (Figure 3). However, the total abundance of the taxon (across different strains) was significantly negatively associated with TLR activation, indicating that increased abundance in general was associated with reduced TLR activation.

## 3 Discussion

The present study characterised the composition of the bacterial microbiome at contemporary waste sorting plants and provided insights in the immune modulating potential of work environmental samples under in vitro conditions. By combining high‐throughput sequencing (HTS) with TLR activation assays, we demonstrated that work‐environmental bacterial communities possessed distinct immune‐modulatory properties, and that a subset of low‐abundance but high ligand‐affine taxa drive most of the observed TLR responses. Significant associations were detected between unique taxa and TLR activation, including both stimulatory and inhibitory relationships. Furthermore, divergent associations between microbial abundance and TLR activation were identified. These findings advance our understanding of the interactions between the bacterial microbiome and innate immunity and assist in enhancing bacteria associated risk evaluations in occupational settings.

Workplace exposure may occur via multiple routes (skin contact, inhalation or ingestion) and across physical and chemical barriers, such as keratinised epithelium, mucous membranes, cilia and enzymatic secretions. Breach of these barriers engages immune cells like monocytes, neutrophils and macrophages, which play a pivotal role as first responders in an innate immune response. These cells are equipped with pattern recognition receptors (PRRs), such as Toll‐like receptors (TLR) that play a pivotal role in modulating immune responses through the interaction with bacterial ligands. TLR activation of monocytes and macrophages causes the synthesis of pro‐inflammatory cytokines and chemokines, whereas activation of neutrophils enhances their affinity and ability to kill pathogens through release of anti‐microbial agents such as reactive oxygen species (ROS) (Hayashi et al. 2003; Schmidt et al. 2025; Riise et al. 2015). The interaction between microbial ligands and TLR on dendritic cells, on the other hand, facilitates cell maturation and migration to lymph cells where antigens are presented to T cells and TLR activation thereby forms a link between the innate and adaptive immune system. Due to the complexity of exposure and exposure‐related immune responses, in vivo inference is difficult. Therefore, in vitro TLR‐specific cell lines remain a useful model to study immunological effects of work‐environmental samples.

The effect of work‐environmental samples on TLR activation has previously been explored to study immunological implications of occupational exposure to bioaerosols. A recent study conducted in the waste management sector has shown a distinct association between TLR activation and work environmental samples, indicating that the work environment contained sufficient microbial ligands capable of inducing an immune response in vitro (Eriksen, Afanou, Straumfors, et al. 2023). However, immune stimulation is affected by the potential of microorganisms to engage TLR receptors.

Microbial components, particularly those rich in lipopolysaccharides derived from Gram‐negative bacteria, such as *E. coli* , *P. haueri* and *P. chlororaphis* , harbour LPS that predominantly engages TLR4 (Amemiya et al. 2021a). In contrast, Gram‐positive bacteria, such as *S. anginosus* , *C. imitans* and *Anaerococcus* spp., primarily signal via TLR2 through lipoproteins/lipoteichoic acid (Kawai and Akira 2010). However, many bacteria, such as *Spirochetes* spp., *Mollicutes* spp.and *Bacteriodota* may still engage TLRs via lipoproteins, atypical lipid A structures or glycosphingolipid‐rich envelopes (Oliveira‐Nascimento et al. 2012; Jenssen et al. 2006; Zubeldia‐Varela et al. 2023; Amemiya et al. 2021b). Previous research has shown that environments rich in endotoxin‐producing bacteria, such as *E. coli*, were associated with strong TLR4‐mediated immune responses compared to work environments that were dominated by microorganisms with low immune stimulatory potential (El‐Zayat et al. 2019a). The present study showed that 71% (TLR2) and 63% (TLR4) of significant responses were associated with Gram‐negative taxa, versus 25% and 33% with Gram‐positives, respectively (Figures 1 and 2), indicating that receptors encountered multiple bacterial ligands dominated by lipopolysaccharides, in mixture with lipoproteins, peptidoglycans and lipoteichoic acid. Surprisingly, all taxa within the phylum Actinobacteriota and Firmicutes, both consisting of Gram‐positive taxa, showed a strong association with positive TLR4 activation. These results suggest alternative pathways or indirect mechanisms of TLR4 activation in vitro, such as interaction with LPS or other cell wall components that can interact with TLR4, either directly or through host‐derived mediators (Silva Lagos et al. 2022). Research on inflammatory diseases has shown that TLR responses seem to be a key regulator for innate immunity (Cario and Podolsky 2000) and vary greatly between bacterial species, and even strains within the same species (El‐Zayat et al. 2019b).

In vitro studies have investigated the associations between the total stimulatory potential of microorganisms; however, so far only a few studies have investigated TLR activation in association with the microbiome and identified bacteria that induced TLR activation at high taxonomic resolution (Amemiya et al. 2021a; Michels et al. 2018). The present study identified 87 unique bacterial taxa belonging to 10 different phyla that were significantly associated with TLR activation. These taxa comprised 296 unique ASV which exerted inconsistent effects on TLR activation, indicating highly variable immune stimulatory potential within a taxon (Figures 3 and S7).

The present study showed that approximately 8% of the ASVs that were identified at species level (1% of the total ASVs in the dataset) contributed measurably to TLR activation, supporting the hypothesis that few strains with high potential to induce TLR activation dominate signalling over abundant but weakly stimulatory taxa. This pattern aligns with taxa such as representatives within the genus *Staphylococci*, which are known to express high‐affinity PAMPs even at low concentrations (Wolf et al. 2011; Ip et al. 2010) as well as with studies showing that ligand processing can amplify TLR activation in sequential waves due to bacterial degradation (Wolf et al. 2011).

Negative associations likely reflect taxa with weak/atypical PAMPs or strategies that dampen TLR signalling, such as direct pathway inhibition, altered lipid A chemistry, or competitive ligand binding (Kondo et al. 2012; Hamerman et al. 2016; Lucas and Maes 2013). CpG‐rich bacterial taxa, such as *Desulfovibrio desulfuricans and Bacteroides* spp. (Figure 3) were strongly, negatively associated with TLR activation in the present study. This is consistent with attenuated TLR4 activity from atypical lipid A and possible promotion of tolerogenic signals (Jenssen et al. 2006; Dalpke et al. 2006).

As with prior bioaerosol studies, the present study observed stronger TLR activation in samples with lower richness (Figure S1), consistent with enrichment for highly inductive taxa and echoing divergent associations reported in fungal exposure research (Afanou et al. 2023). In the present study, high TLR activation was generally associated with low‐abundance species, supporting the findings of Afanou and colleagues, who identified divergent associations between fungal diversity as well as fungal spore concentrations and in vitro TLR activation. In fact, negative associations between fungal richness and TLR activation indicate that TLR receptors have more affinity for few but highly potent pathogenic ligands, rather than highly abundant ligands derived from bacteria with low immune stimulatory potential. Divergent trends were also observed within *Anaerococcus vaginalis* (Figure S8), where two low‐abundance ASVs displayed distinct effects, with ASV 1 being associated with relatively high activation and ASV 2 exhibiting variable activation intensity.

It must be considered that in vitro systems simplify exposure and typically lack phagocytic processing and a dynamic cytokine milieu. Phagocytosis can profoundly shape TLR signalling by affecting uptake, endosomal trafficking, and ligand processing that may expose otherwise cryptic PAMPs and generate secondary waves of activation in endosomal compartments (Underhill and Goodridge 2012). Complex mixtures may produce synergy or antagonism amongst ligands (e.g., *Bacteroidota* lipid A antagonism against TLR4), and strain‐level heterogeneity further complicates inference (Kawai and Akira 2010; Wolf et al. 2011). Thus, receptor activation in vivo reflects both the cell intrinsic phagocytic capacity and the kinetics of ligand processing, which our cell line model does not capture. This may, in part, explain the dominance of low‐abundant, highly‐inductive taxa in the cell model, whereas in vivo these same ligands could be amplified, reshaped or curtailed by phagocytosis and compartmentalised TLR engagement (Underhill and Goodridge 2012).

Cytokine autocrine and paracrine loops further modulate TLR outputs in vivo. Early TLR2/4 signalling can induce IL 1β, TNFα and type I interferons, which then feedback via NFκB/IRF and JAK/STAT pathways to amplify or reprogramme receptor responses in the same (autocrine) or neighbouring (paracrine) cells (Mudaliar et al. 2014). Such loops can heighten sensitivity to weak ligands, dampen responses through negative regulators, or shift the balance between TLR2 and TLR4 pathways depending on context (Caldwell et al. 2014). The reporter system used in the present study measures primarily receptor activation in the absence of these forward/feedback circuits; hence, the magnitude and even direction of TLR readouts observed here may differ under physiological autocrine conditions where cytokine crosstalk can potentiate or constrain signalling. Autocrine amplification also offers a plausible explanation for unexpected positive associations between some Gram‐positive taxa and TLR4 in vivo: primary TLR2 engagement by lipoproteins/LTA may elicit TNFα/IL 1β, which secondarily primes TLR4 pathways and co‐stimulation (Bösl et al. 2018). Whilst our in vitro model revealed these taxa as TLR4 associated in mixed samples, we interpret this cautiously as a likely consequence of co‐extracted LPS and the absence of cytokine driven cross priming in the assay.

Nevertheless, in vitro studies help dissect microbial effects on TLR without the complexity of in vivo systems and thereby aid the understanding of underlying molecular mechanisms in a simplified model system; however, as shown in the present study, only a minor fraction of the microbiome drove activation, underscoring the gap to in vivo complexity. Differential TLR activation by low‐abundant, highly inductive bacteria versus abundant, weakly inductive can shape downstream biology and health effects (El‐Zayat et al. 2019b). Potent activators promote pathogen clearance via IL‐6 and TNFα, yet can also precipitate excessive inflammation, including cytokine storm and acute respiratory distress syndrome (ARDS) (Aisiku et al. 2016). Integrating microbiome profiles with TLR readouts pinpoints driver taxa relevant to occupational risk. In waste sorting plants, targeting LPS‐rich Gram‐negatives (e.g., *Escherichia–Shigella*, *Proteus*, *Pseudomonas*, *Wohlfahrtiimonas*) and TLR2‐potent Gram‐positives (e.g., *Streptococcus*, *Corynebacterium*, *Anaerococcus*) may better mitigate inflammatory burden than focusing on total load alone, whilst recognising taxa that dampen TLR signalling (e.g., *Bacteroides*, *Desulfovibrio*) refines interpretation of mixed exposures. Overall, coupling microbiome data with in vitro assays supports risk assessment and exposure reduction strategies for immune‐stimulatory microorganisms in occupational settings. However, to better approximate in vivo conditions, future studies should employ advanced cell models that capture the dynamics of TLR signalling.

## 4 Conclusion

The present study revealed convergent associations between bacterial abundance and TLR activation, suggesting that low abundant, ligand‐inducing taxa disproportionally drive TLR signalling in vitro, whilst the bulk of the bacterial community contributes little to receptor activation. Consistent with this, in vitro assays showed that only about 8% of the bacterial microbiome induced measurable TLR signalling. Both Gram‐positive and Gram‐negative taxa were associated with TLR2 and TLR4 activation across cell line, with notable inhibitory signals amongst Bacteriodota, and several unique taxa exhibited inhibitory effects on receptor activation. Together, these findings support ligand‐centric, taxon‐aware risk assessment strategies in occupational environments.

### 4.1 Shortcomings of the Study

Amplicon sequencing targeted V4/V5 16S rRNA regions, which can introduce primer bias and uneven taxon representation. In vitro models, whilst mechanistically informative, lack phagocytic processing and tissue context, limiting generalisability to in vivo conditions. Sample size and strain‐level heterogeneity further constrain inference.

## 5 Material and Methods

This study used personal full‐shift air samples that were collected at six contemporary Norwegian waste sorting plants in the period June 2020 until November 2021. A total of 55 workers participated in the study and helped retrieve 110 personal full‐shift samples for the analysis of the inhalable microbiome (*n* = 55) and in vitro cell experiments (*n* = 55) to study the associations between bacterial community composition and the inflammatory potential in work air samples. Sampling, DNA sequencing and in vitro experiments were carried out as described elsewhere (Eriksen, Afanou, Straumfors, et al. 2023; Eriksen, Madsen, Afanou, et al. 2023), however, only sequenced samples with corresponding TLR activation data were included in the downstream analysis.

### 5.1 Data Analyses

All data analyses were executed in R/R Studio version 4.4.1.

This manuscript was developed without the use of artificial Intelligence Generated Content (AIGC) tools.

ASVs were identified as previously described by Eriksen, Afanou, Straumfors, et al. (2023). Diversity analyses were performed using phyloseq (McMurdie and Holmes 2013) and vegan (Oksanen 2022). Gram‐positive and negative species as well as pathogenicity of taxa were assigned using the AMR package (Berends et al. 2022). Graphics were produced in ggplot2 (Wickham 2016). Compositional data analysis was performed using compositions (van den Boogaart et al. 2024). In vitro *exposure* experiments were executed in biological replicates as described in Eriksen, Afanou, Straumfors, et al. (2023). Mean absorbance levels of biological parallels corrected for absorbance of negative controls were used in further analyses.

After filtering steps, the dataset included 21,747 unique high‐quality ASVs. Two thousand six hundred sixty‐three unique ASVs that were identified at the species level were included in the downstream analysis and subsequently aggregated at species level resulting in 1110 unique taxa. Taxa with zero counts were replaced by 0.5 prior to transformation to relative abundance (Sisk‐Hackworth and Kelley 2020) and centre log transformation.

Correlation (Spearman) between bacterial richness (ACE) and diversity (Shannon), respectively and TLR activation as well as between unique taxa and TLR activation in vitro were reported. Correlations with a *p*‐value below 0.05 (two‐tailed) were considered statistically significant. Bacterial taxa with statistically significant correlation to TLR activation were presented in the results and used in further analysis. A list of all identified ASVs, *p*‐values as well as correlation coefficients can be found in the paper supplement.

Abundance of taxa was reported for samples that were associated with significant activation of TLR2 (*n* = 18) and TLR4 (*n* = 18), respectively in comparison to a parental HEK null cell line. *p*‐values below 0.05 were considered statistically significant. For further details on samples and in vitro experiments consult.

## Author Contributions

**Elke Eriksen:** conceptualization, data curation, formal analysis, investigation, methodology, resources, validation, visualization, writing – review and editing, writing – original draft. **Pål Graff:** conceptualization, funding acquisition, project administration, writing – review and editing. **Anani Komlavi Afanou:** conceptualization, supervision, writing – review and editing.

## Funding

This work was financially supported by the National institute of occupational health, Oslo (STAMI); IVAR‐IKS; The federation of Norwegian Industries; and the Norwegian Union of Municipal and General Employees (NUMEG).

## Ethics Statement

The study was approved by the Regional Ethics Committee in Oslo, REC South‐East B (ref. no.: 34312) and written consent was obtained prior to participation.

## Conflicts of Interest

The authors declare no conflicts of interest.

## Supporting information


**Data S1:** emi470289‐sup‐0001‐Supinfo.pdf.

## Data Availability

The data that support the findings of this study are available on request from the corresponding author. The data are not publicly available due to privacy or ethical restrictions.
