# Supplementary material for: Bacterial Communities as Modulators of Innate Immune Signalling: An In Vitro Perspective on Toll‐Like Receptor Activation
Source: Environ Microbiol Rep. 2026 Feb 4;18(1):e70289. doi: 10.1111/1758-2229.70289 (PMC12872116; doi:10.1111/1758-2229.70289)
Supplement: Supplementary file 1 — Data S1: emi470289‐sup‐0001‐Supinfo.pdf. [file EMI4-18-e70289-s001.pdf]

Supporting Information for

Bacterial communities as modulators of innate immune signalling: an *in vitro* perspective on Toll-like receptor activation

Elke Eriksen*[1], Pål Graff[1], Anani Komlavi Afanou[2]

[1] STAMI, National Institute of Occupational Health, Research Group for Chemical Work Environment, Gydas Vei 8, 0363 Oslo, Norway

[2] STAMI, National Institute of Occupational Health, Research Group for Occupational Toxicology, Gydas Vei 8, 0363 Oslo, Norway

* Email: elke.eriksen@stami.no

This PDF file includes:

Supporting figures 1S to 8S

Supporting tables 1S to 2S

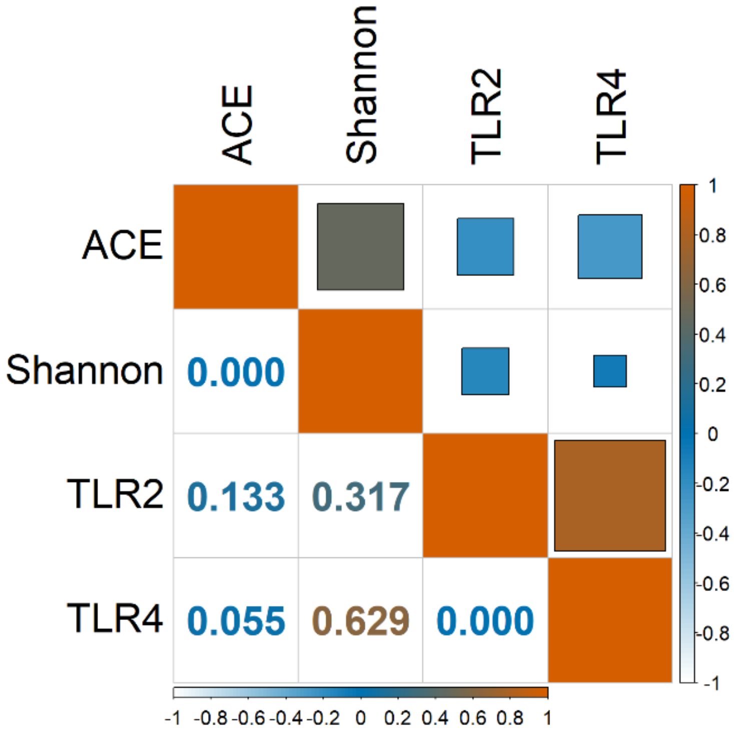

Figure 1S - Correlation analysis of ACE and Shannon diversity indices and TLR2 and TLR4 activation. Upper corner: Shannon correlation coefficient indicated in colour gradient, lower corner: Benjamini Hochberg corrected p values.

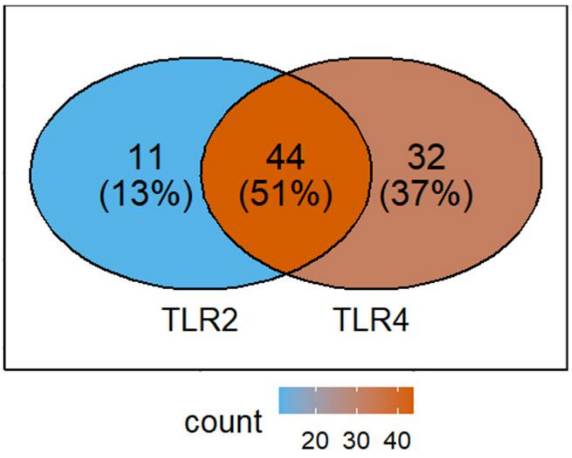

Figure 2S - Venn diagram of shared taxa that significantly correlated with TLR2 and TLR4 activation.

Table 1S - Taxa associated with TLR2 and TLR4 activation measured in the in vitro model, information on established Pathogen-associated molecular patterns (PAPMs) and occupational/pathogenic relevance for humans. Most relevant human pathogens are highlighted in blue.

	TLR2 PAMPs (typical)	TLR4 PAMPs (typical)	Human health relevance	Occupational exposure relevance	Ref (author, year, DOI)
Acetobacter cibinongensis / -	Lipoproteins, porins	LPS (lipid A)	Environmental vinegar bacteria; rare human disease	Food fermentation workers; aerosolized endotoxin risk	
Acetobacter lovaniensis / -	Lipoproteins	LPS	Environmental; rare	Food/beverage fermentation facilities	
Akkermansia muciniphila / -	Outer membrane protein Amuc_1100 (TLR2), lipoproteins	LPS (low-immunogenic)	Gut commensal, metabolic benefits	Lab animal facilities; probiotics manufacturing	Aggarwal et al, 2022, 10.1007/s12223-022-00973-6
Alistipes indistinctus / -	Lipoproteins	LPS	Gut commensal; dysbiosis links	Faecal handling	
Anaerococcus nagyae / +	LTA, lipoproteins, peptidoglycan	—	Opportunistic (abscesses)	Healthcare	
Anaerococcus prevotii / +	LTA, lipoproteins, peptidoglycan	—	Opportunistic anaerobe	Healthcare, dental	
Anaerococcus vaginalis / +	LTA, lipoproteins	—	BV-associated	Healthcare	
Anaerostipes hadrus / +	LTA, peptidoglycan	—	Butyrate-producing gut commensal		
Anoxybacillus flavithermus / +	LTA, lipoproteins	—	Environmental thermophile		
Bacteroides caccae / -	Lipoproteins	LPS (variable immunogenicity)	Gut commensal; opportunistic	Faecal labs; healthcare	Wang, et al, 2021, 10.1080/10408398.2020.1802695<br>Yang, et al, 2021, 10.1177/03000605211047277
Bacteroides coprocola / -	Lipoproteins	LPS	Gut commensal	Fecal handling	
Bacteroides helcogenes / -	Lipoproteins	LPS	Animal/human gut	Veterinary, pet dentistry	
Bacteroides massiliensis / -	Lipoproteins	LPS	Gut commensal		
Bacteroides propionicifaciens / -	Lipoproteins	LPS	Gut commensal		
Bacteroides reticulotermitis / -	Lipoproteins	LPS	Insect gut; minimal human relevance		
Blastopirellula marina / -	Lipoproteins	LPS	Marine; minimal	Aquaculture	
Blautia glucerasea / +	LTA, peptidoglycan	—	Beneficial gut commensal		
Brevundimonas subvibrioides / -	Lipoproteins	LPS	Opportunistic in immunocompromised	Healthcare water systems; lab water	
Brumimicrobium glaciale / -	Lipoproteins	LPS	Psychrophile; minimal	Polar/marine research	
Burkholderia-Caballeronia-Paraburkholderia pseudomallei / -	Lipoproteins	LPS (unusual lipid A), CPS	Melioidosis; severe systemic disease	Water exposure; BSL-3 labs	Sanchez-Villamil et al, 2021, 10.1128/IAI.00654-20
Burkholderia-Caballeronia-Paraburkholderia tropica / -	Lipoproteins	LPS	Plant-associated; rare human disease	Agricultural biocontrol; soil exposure	
Chryseobacterium glaciei / -	Lipoproteins	LPS	Environmental; rare		
Chryseobacterium halperniae / -	Lipoproteins	LPS	Opportunistic	Hospital water systems	

Chryseobacterium hispanicum / -	Lipoproteins	LPS	Rare	Water systems	
Chryseobacterium soldanellicola / -	Lipoproteins	LPS	Environmental		
Corynebacterium imitans / +	LTA, lipoproteins	—	Skin/oropharyngeal; opportunistic	Healthcare	Qiu et al, 2023, 10.1177/11769343231191481
Desulfovibrio desulfuricans / -	Lipoproteins	LPS	Opportunistic bacteria	Wastewater, oil/gas, sewer workers	
Devosia riboflavina / -	Lipoproteins	LPS	Environmental	Soil/water	
Dyadobacter fermentans / -	Lipoproteins	LPS	Environmental	Soil/microbiome	
Dysgonomonas capnocytophagoides / -	Lipoproteins	LPS	Opportunistic, GI	Healthcare	
Erysipelotrichaceae UCG-003 bacterium / +	LTA, peptidoglycan	—	Gut-associated; dysbiosis links		
Escherichia-Shigella coli / -	Lipoproteins	LPS (TLR4)	Major pathogen/commensal	Healthcare, wastewater; labs; food handling	Silva et al, 2024, 10.3390/pathogens13040305
Flavobacterium saccharophilum / -	Lipoproteins	LPS	Environmental	Aquaculture	
Flavobacterium tegetincola / -	Lipoproteins	LPS	Environmental	Aquatic systems	
Fusicatenibacter saccharivorans / +	LTA, peptidoglycan	—	Beneficial gut bacterium		
Gluconobacter frateurii / -	Lipoproteins	LPS	Food-associated; rare	Fermentation industries	
Hymenobacter elongatus / -	Lipoproteins	LPS	Environmental		
Hymenobacter qilianensis / -	Lipoproteins	LPS	Environmental		
Hymenobacter tibetensis / -	Lipoproteins	LPS	Environmental		
Hymenobacter xinjiangensis / -	Lipoproteins	LPS	Environmental		
Hyphomicrobium facile / -	Lipoproteins	LPS	Environmental methylotroph	Wastewater treatment plants	
Koukoulia aurantiaca / -	Lipoproteins	LPS	Environmental		
Lachnospiraceae NK4A136 group bacterium / +	LTA, peptidoglycan	—	Beneficial gut commensal		
Lysinibacillus xylanilyticus / +	LTA, lipoproteins	—	Environmental; rare	Soil/industrial enzyme production	
Myroides guanonis / -	Lipoproteins	LPS (unusual)	Opportunistic	Healthcare; wastewater	
Myroides odoratus / -	Lipoproteins	LPS	Opportunistic	Healthcare	Xu et al, 2018, 10.1089/mdr.2017.0233
Myroides phaeus / -	Lipoproteins	LPS	Opportunistic	Similar exposures	
Myroides profundi / -	Lipoproteins	LPS	Deep-sea; minimal	Marine labs	
Nitrospira defluvii / -	Lipoproteins	LPS	Environmental nitrifier	Wastewater treatment; bioaerosols	
Paenibacillus tumbae / +	LTA, lipoproteins	—	Environmental; rare	Soil/agriculture	
Parabacteroides johnsonii / -	Lipoproteins	LPS	Gut commensal		
Pectobacterium parmentieri / -	Lipoproteins	LPS	Plant pathogen; rare human	Agriculture	

Pediococcus pentosaceus / +	LTA, peptidoglycan	—	Food fermenter; rare opportunist	Food fermentation workers	
Pedobacter terrae / -	Lipoproteins	LPS	Environmental	Soil labs	
Peptoniphilus obesi / +	LTA, peptidoglycan	—	Skin/soft tissue/gynaecologic	Healthcare	
Porphyromonas cangingivalis / -	Lipoproteins	LPS (atypical lipid A)	Animal oral pathogen	Veterinary, pet dentistry	
Prevotella bivia / -	Lipoproteins	LPS	Vaginal/obstetric infections	Healthcare; gynaecology	Muzny et al, 2020, 10.1097/QCO.0000000000000620
Prevotella buccalis / -	Lipoproteins	LPS	Oral flora; periodontal	Dentistry; oral surgery	
Prevotella corporis / -	Lipoproteins	LPS	Oral/soft tissue infections	Dentistry; healthcare	
Prevotella nanceiensis / -	Lipoproteins	LPS	Respiratory/oral	Healthcare	
Prevotella salivae / -	Lipoproteins	LPS	Oral flora; periodontal	Dentistry	
Prevotella_7 pleuritidis / -	Lipoproteins	LPS	Respiratory infections	Healthcare	
Proteus hauseri / -	Lipoproteins	LPS	Catheter-associated infections	Healthcare	Drzewiecka, 2016, 10.1007/s00248-015-0720-6
Pseudoclostridium thermosuccinogenes / +	LTA, peptidoglycan	—	Environmental thermophile	Industrial bioprocessing	
Pseudomonas chlororaphis / -	Lipoproteins	LPS	Environmental biocontrol; rare	Agriculture/greenhouse workers	
Pseudomonas frederiksbergensis / -	Lipoproteins	LPS	Environmental	Soil/bioremediation	
Roseomonas frigidaquae / -	Lipoproteins	LPS	Opportunistic; pink-pigmented	Hospital water, dialysis units	
Sphingomonas astaxanthinifaciens / -	Glycosphingolipids (TLR2)	LPS absent/weak	Environmental; low pathogenicity	Water systems; pigment biotech	
Sphingomonas azotifigens / -	Glycosphingolipids (TLR2)	No LPS	Environmental	Water/soil fieldwork	
Spiroplasma ixodetis / +	Lipoproteins (TLR2)	—	Tick-associated; emerging	Field entomology, veterinary labs	
Spirosoma spitsbergense / -	Lipoproteins	LPS	Environmental		
Sporosarcina pasteurii / +	LTA, peptidoglycan	—	Environmental urease-producer	Construction/biocement	
Streptococcus anginosus / +	LTA, peptidoglycan	—	Abscess-former; invasive	Healthcare, dentistry	Rahman et al, 2015, 10.1128/genomeA.01440-15
Streptococcus dysgalactiae / +	LTA, peptidoglycan	—	Zoonotic soft tissue	Animal handling, dairy farms	Rantala, 2014, 10.1007/s10096-014-2092-0
Streptococcus oralis / +	LTA, peptidoglycan	—	Oral commensal; endocarditis	Dentistry; healthcare	
Streptococcus parauberis / +	LTA, peptidoglycan	—	Fish pathogen; rare human	Aquaculture workers	
Streptococcus urinalis / +	LTA, peptidoglycan	—	UTI cases	Healthcare	
Streptococcus vestibularis / +	LTA, peptidoglycan	—	Oral commensal; opportunistic	Dentistry; healthcare	
Tepidimicrobium ferriphilum / +	LTA, peptidoglycan	—	Thermophile; minimal	Industrial bioprocess	
Terriglobus roseus / -	Lipoproteins	LPS	Acidobacteria; environmental	Soil	

Thermogutta terrifontis / -	Lipoproteins	LPS	Planctomycete; minimal	Hot spring research	
Tumebacillus ginsengisoli / +	LTA, peptidoglycan	—	Environmental; rare	Agricultural soils	
Turneriella parva / -	Lipoproteins (TLR2)	LPS atypical/low	Environmental spirochete	Water/soil fieldwork	
Varibaculum cambriense / +	LTA, peptidoglycan	—	Abscesses/soft tissue	Healthcare	
Vicinamibacter silvestris / -	Lipoproteins	LPS	Environmental	Soil	
Wautersiella falsenii / -	Lipoproteins	LPS	Opportunistic; MDR	Healthcare settings; water systems	
Wohlfahrtiimonas chitiniclastica / -	Lipoproteins	LPS	Wound infections linked to myiasis	Waste management, forensic, pest control	Karaca et al, 2022, 10.14744/tjtes.2022.01409

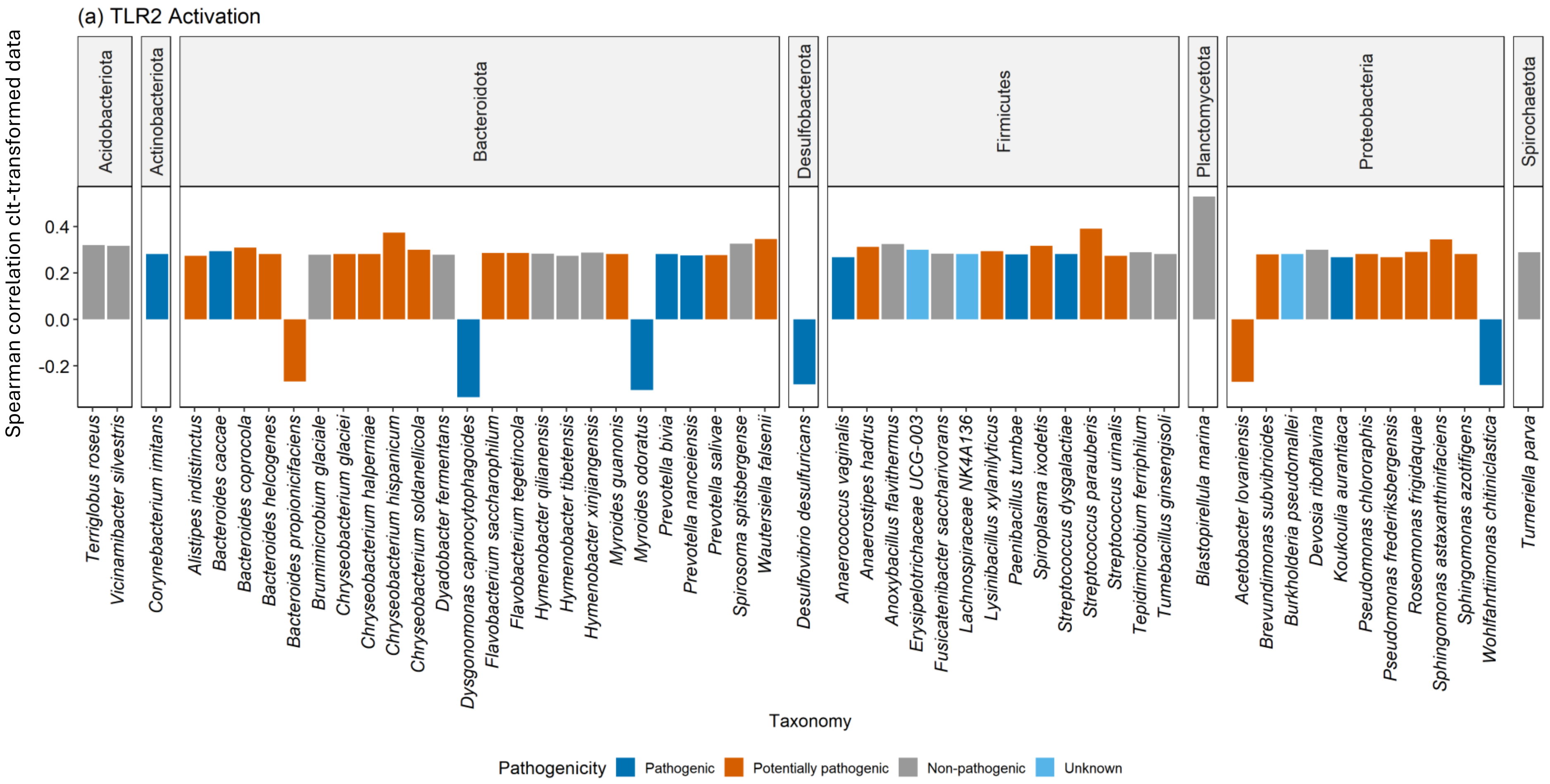

Figure 3S - Correlation between unique taxa and TLR2 activation in vitro (Spearman correlation coefficient, p-value < 0.05). Human pathogens (blue: 22%), Potential pathogens (orange: 45%), non-pathogenic and unknown (grey and light blue: 33%). The correlations for individual taxa are listed in Table 2S.

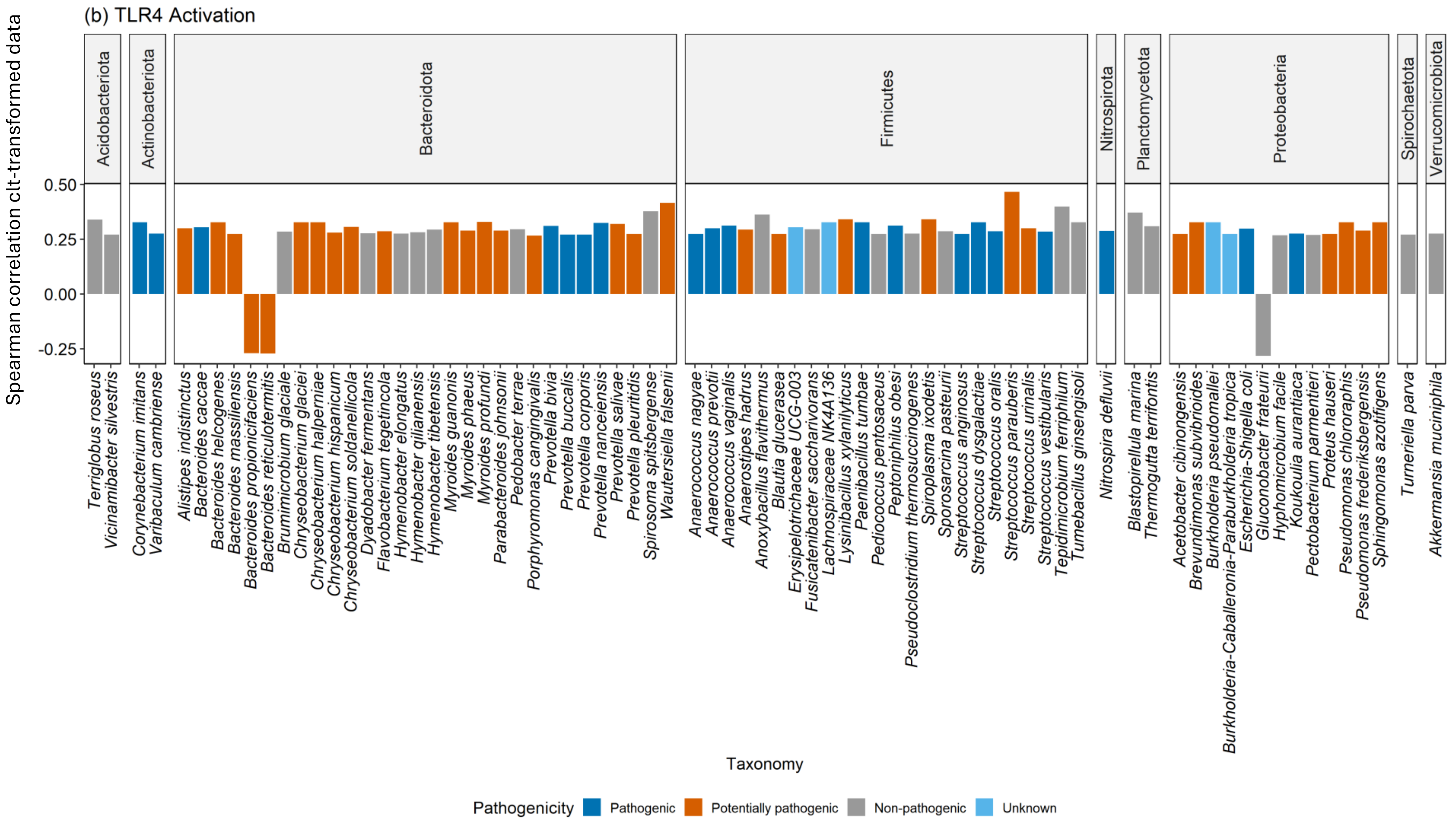

Figure 4S – Correlation between unique taxa and TLR4 activation in vitro (Spearman correlation coefficient, p-value < 0.05). Human pathogens (blue: 25%), Potential pathogens (orange: 37%), non-pathogenic and unknown (grey and light blue: 38%). The correlations for individual taxa are listed in Table 2S.

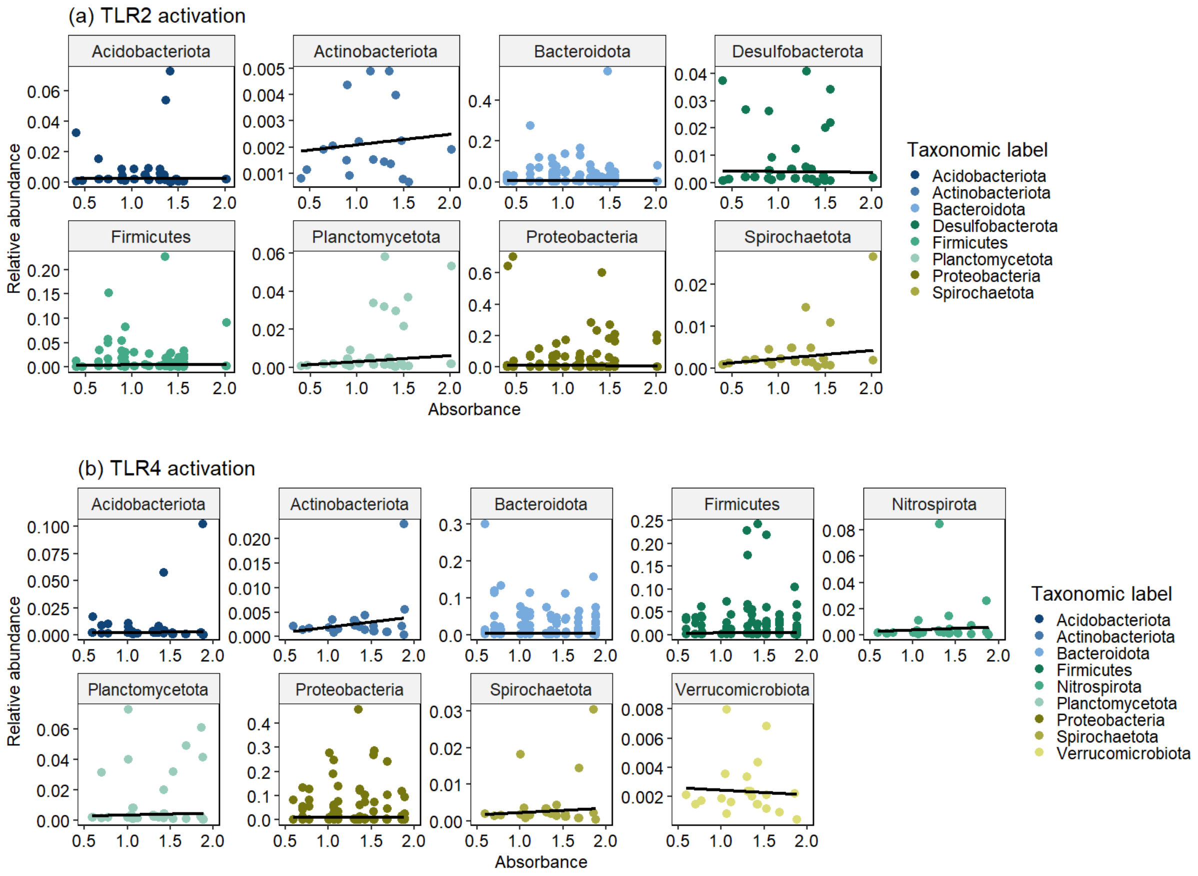

Figure 5S - TLR activation in relation to relative abundance of taxa. Regression lines within Phylum er indicated as solid blackline. (a) TLR2 activation, (b) TLR4 activation. The correlations for individual taxa are listed in Table 2S.

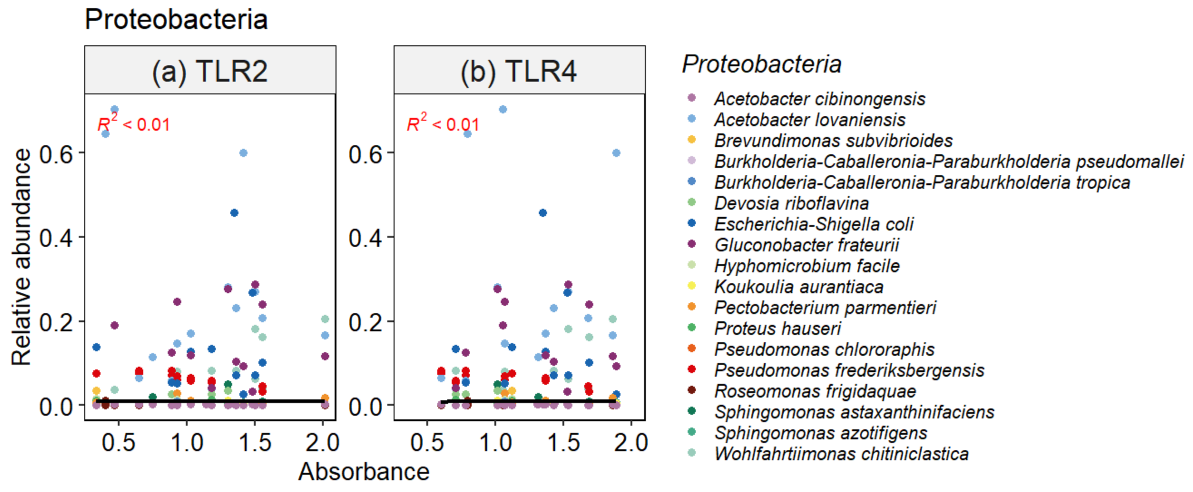

Figure 6S - Associations between TLR activation and unique ASVs. The graph shows samples that significantly activated TLR compared to a paternal HEK null cell li ne. (a) TLR2, (b) TLR4. R^2 of global linear regression given in upper left corner. The correlations for individual taxa are listed in Table 2S.

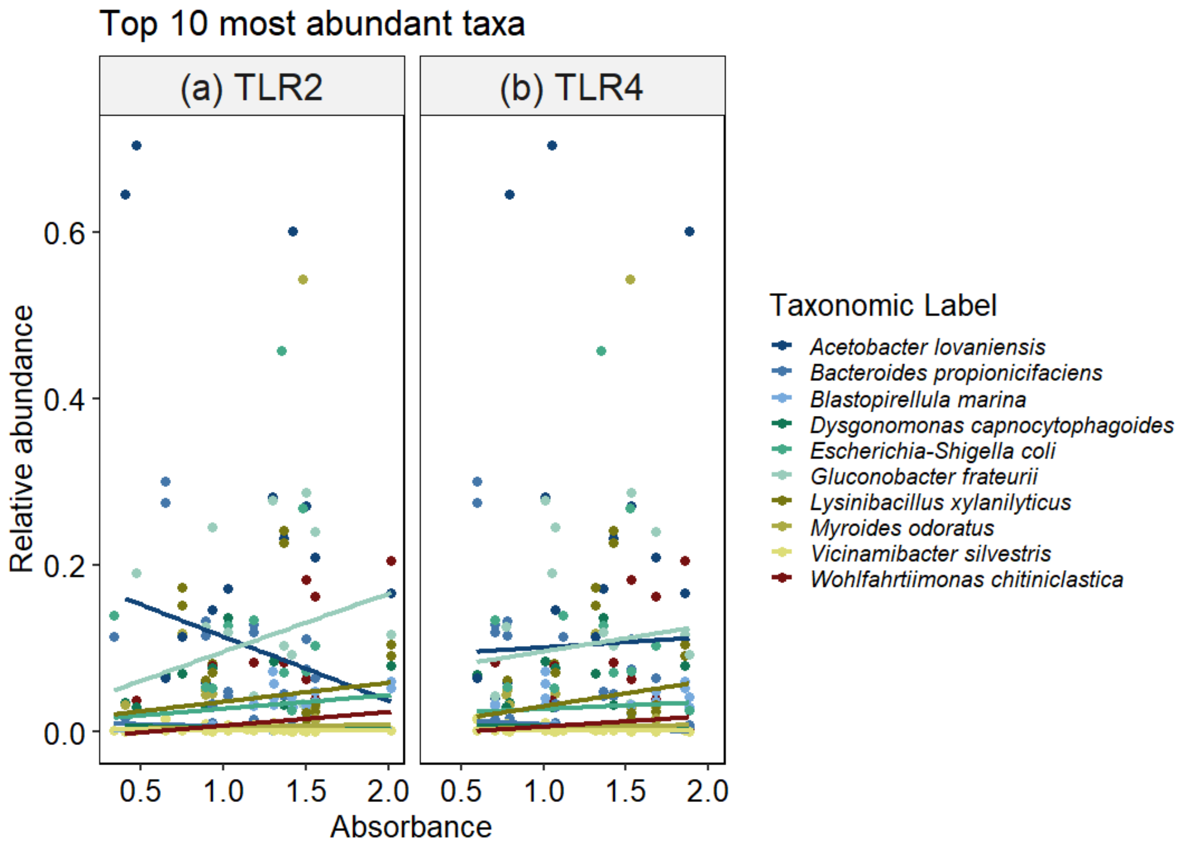

Figure 7S - Associations between TLR activation and top abundant taxa stratified by cell line. The graph shows samples that significantly activated TLR compared to a paternal HEK null cell li ne. (a) TLR2, (b) TLR4. The correlations for individual taxa are listed in Table 2S.

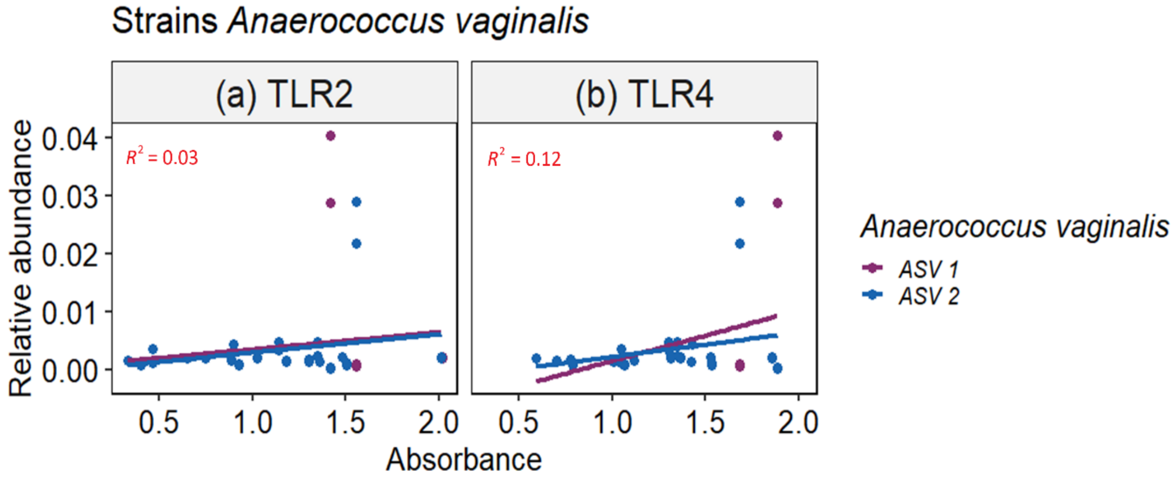

Figure 8S - Associations between TLR activation and ASVs in the taxa A. vaginalis. The graph shows samples that significantly activated TLR compared to a paternal HEK null cell line. (a) TLR2, (b) TLR4. $R^2$of the global regression line across strains indicated in the upper left corner. The correlations for individual taxa are listed in Table 2S.

Table 2S - Correlation between TLR2 and TLR 4 activation and unique taxa. Spearman correlation coefficients and p values above 0.05 included.

	Correlation	p value	*Taxon*
TLR2 activation	-0.2693	0.0471	*Acetobacter lovaniensis*
	0.2732	0.0439	*Alistipes indistinctus*
	0.2680	0.0482	*Anaerococcus vaginalis*
	0.3120	0.0208	*Anaerostipes hadrus*
	0.3242	0.0161	*Anoxybacillus flavithermus*
	0.2930	0.0303	*Bacteroides caccae*
	0.3082	0.0224	*Bacteroides coprocola*
	0.2807	0.0382	*Bacteroides helcogenes*
	-0.2672	0.0489	*Bacteroides propionicifaciens*
	0.5284	0.0000	*Blastopirellula marina*
	0.2797	0.0390	*Brevundimonas subvibrioides*
	0.2786	0.0397	*Brumimicrobium glaciale*
	0.2807	0.0382	*Burkholderia-Caballeronia-Paraburkholderia pseudomallei*
	0.2807	0.0382	*Chryseobacterium glaciei*
	0.2807	0.0382	*Chryseobacterium halperniae*
	0.3730	0.0053	*Chryseobacterium hispanicum*
	0.3001	0.0264	*Chryseobacterium soldanellicola*
	0.2807	0.0382	*Corynebacterium imitans*
	-0.2795	0.0391	*Desulfovibrio desulfuricans*
	0.2994	0.0267	*Devosia riboflavina*
	0.2784	0.0400	*Dyadobacter fermentans*
	-0.3356	0.0126	*Dysgonomonas capnocytophagoides*
	0.2996	0.0266	*Erysipelotrichaceae UCG-003 bacterium*
	0.2866	0.0343	*Flavobacterium saccharophilum*
	0.2852	0.0352	*Flavobacterium tegetincola*
	0.2826	0.0369	*Fusicatenibacter saccharivorans*
	0.2832	0.0365	*Hymenobacter qilianensis*
	0.2737	0.0435	*Hymenobacter tibetensis*
	0.2875	0.0337	*Hymenobacter xinjiangensis*
	0.2670	0.0491	*Koukoulia aurantiaca*
	0.2807	0.0382	*Lachnospiraceae NK4A136 group bacterium*
	0.2933	0.0301	*Lysinibacillus xylanilyticus*
	0.2807	0.0382	*Myroides guanonis*
	-0.3048	0.0241	*Myroides odoratus*
	0.2804	0.0385	*Paenibacillus tumbae*
	0.2811	0.0380	*Prevotella bivia*
	0.2759	0.0418	*Prevotella nanceiensis*
	0.2769	0.0410	*Prevotella salivae*
	0.2807	0.0382	*Pseudomonas chlororaphis*
	0.2669	0.0492	*Pseudomonas frederiksbergensis*

	0.2910	0.0315	*Roseomonas frigidaquae*
	0.3449	0.0102	*Sphingomonas astaxanthinifaciens*
	0.2807	0.0382	*Sphingomonas azotifigens*
	0.3162	0.0190	*Spiroplasma ixodetis*
	0.3254	0.0157	*Spirosoma spitsbergense*
	0.2807	0.0382	*Streptococcus dysgalactiae*
	0.3904	0.0034	*Streptococcus parauberis*
	0.2732	0.0439	*Streptococcus urinalis*
	0.2897	0.0323	*Tepidimicrobium ferriphilum*
	0.3198	0.0177	*Terriglobus roseus*
	0.2807	0.0382	*Tumebacillus ginsengisoli*
	0.2887	0.0329	*Turneriella parva*
	0.3174	0.0186	*Vicinamibacter silvestris*
	0.3455	0.0101	*Wautersiella falsenii*
	-0.2828	0.0368	*Wohlfahrtiimonas chitiniclastica*
**TLR4 activation**	0.2737	0.0432	*Acetobacter cibinongensis*
	0.2762	0.0412	*Akkermansia muciniphila*
	0.3007	0.0257	*Alistipes indistinctus*
	0.2737	0.0432	*Anaerococcus nagyae*
	0.3000	0.0261	*Anaerococcus prevotii*
	0.3122	0.0203	*Anaerococcus vaginalis*
	0.2935	0.0296	*Anaerostipes hadrus*
	0.3625	0.0065	*Anoxybacillus flavithermus*
	0.3044	0.0239	*Bacteroides caccae*
	0.3277	0.0146	*Bacteroides helcogenes*
	0.2739	0.0430	*Bacteroides massiliensis*
	-0.2696	0.0466	*Bacteroides propionicifaciens*
	-0.2714	0.0450	*Bacteroides reticulotermitis*
	0.3719	0.0052	*Blastopirellula marina*
	0.2745	0.0425	*Blautia glucerasea*
	0.3271	0.0148	*Brevundimonas subvibrioides*
	0.2846	0.0352	*Brumimicrobium glaciale*
	0.3277	0.0146	*Burkholderia-Caballeronia-Paraburkholderia pseudomallei*
	0.2737	0.0432	*Burkholderia-Caballeronia-Paraburkholderia tropica*
	0.3277	0.0146	*Chryseobacterium glaciei*
	0.3277	0.0146	*Chryseobacterium halperniae*
	0.2798	0.0386	*Chryseobacterium hispanicum*
	0.3069	0.0227	*Chryseobacterium soldanellicola*
	0.3277	0.0146	*Corynebacterium imitans*
	0.2766	0.0410	*Dyadobacter fermentans*
	0.3049	0.0236	*Erysipelotrichaceae UCG-003 bacterium*
	0.2983	0.0269	*Escherichia-Shigella coli*
	0.2860	0.0343	*Flavobacterium tegetincola*

0.2961	0.0282	*Fusicatenibacter saccharivorans*
-0.2820	0.0370	*Gluconobacter frateurii*
0.2755	0.0418	*Hymenobacter elongatus*
0.2820	0.0370	*Hymenobacter qilianensis*
0.2940	0.0293	*Hymenobacter tibetensis*
0.2681	0.0478	*Hyphomicrobium facile*
0.2754	0.0419	*Koukoulia aurantiaca*
0.3277	0.0146	*Lachnospiraceae NK4A136 group bacterium*
0.3413	0.0108	*Lysinibacillus xylanilyticus*
0.3277	0.0146	*Myroides guanonis*
0.2894	0.0321	*Myroides phaeus*
0.3295	0.0140	*Myroides profundi*
0.2874	0.0334	*Nitrospira defluvii*
0.3270	0.0148	*Paenibacillus tumbae*
0.2896	0.0320	*Parabacteroides johnsonii*
0.2692	0.0468	*Pectobacterium parmentieri*
0.2737	0.0432	*Pediococcus pentosaceus*
0.2960	0.0282	*Pedobacter terrae*
0.3117	0.0205	*Peptoniphilus obesi*
0.2669	0.0489	*Porphyromonas cangingivalis*
0.3112	0.0208	*Prevotella bivia*
0.2716	0.0449	*Prevotella buccalis*
0.2716	0.0449	*Prevotella corporis*
0.3250	0.0155	*Prevotella nanceiensis*
0.3194	0.0175	*Prevotella salivae*
0.2749	0.0423	*Prevotella_7 pleuritidis*
0.2748	0.0423	*Proteus hauseri*
0.2749	0.0422	*Pseudoclostridium thermosuccinogenes*
0.3277	0.0146	*Pseudomonas chlororaphis*
0.2887	0.0325	*Pseudomonas frederiksbergensis*
0.3277	0.0146	*Sphingomonas azotifigens*
0.3412	0.0108	*Spiroplasma ixodetis*
0.3770	0.0045	*Spirosoma spitsbergense*
0.2858	0.0344	*Sporosarcina pasteurii*
0.2745	0.0425	*Streptococcus anginosus*
0.3277	0.0146	*Streptococcus dysgalactiae*
0.2867	0.0338	*Streptococcus oralis*
0.4666	0.0003	*Streptococcus parauberis*
0.3007	0.0257	*Streptococcus urinalis*
0.2852	0.0348	*Streptococcus vestibularis*
0.3994	0.0025	*Tepidimicrobium ferriphilum*
0.3397	0.0112	*Terriglobus roseus*
0.3088	0.0218	*Thermogutta terrifontis*

0.3277	0.0146	*Tumebacillus ginsengisoli*
0.2710	0.0454	*Turneriella parva*
0.2751	0.0421	*Varibaculum cambriense*
0.2707	0.0456	*Vicinamibacter silvestris*
0.4153	0.0016	*Wautersiella falsenii*
